# Corticomuscular synchronization with small and large dynamic force output

**DOI:** 10.1186/1471-2202-8-101

**Published:** 2007-11-27

**Authors:** Agnieszka Andrykiewicz, Luis Patino, Jose Raul Naranjo, Matthias Witte, Marie-Claude Hepp-Reymond, Rumyana Kristeva

**Affiliations:** 1 Neurological Clinic, University Freiburg, Breisacherstraße 64, 79106 Freiburg, Germany; 2 Institute of Neuroinformatics, University and ETH, Zürich, Germany

## Abstract

**Background:**

Over the last few years much research has been devoted to investigating the synchronization between cortical motor and muscular activity as measured by EEG/MEG-EMG coherence. The main focus so far has been on corticomuscular coherence (CMC) during static force condition, for which coherence in beta-range has been described. In contrast, we showed in a recent study [1] that dynamic force condition is accompanied by gamma-range CMC. The modulation of the CMC by various dynamic force amplitudes, however, remained uninvestigated. The present study addresses this question. We examined eight healthy human subjects. EEG and surface EMG were recorded simultaneously. The visuomotor task consisted in isometric compensation for 3 forces (static, small and large dynamic) generated by a manipulandum. The CMC, the cortical EEG spectral power (SP), the EMG SP and the errors in motor performance (as the difference between target and exerted force) were analyzed.

**Results:**

For the static force condition we found the well-documented, significant beta-range CMC (15–30 Hz) over the contralateral sensorimotor cortex. Gamma-band CMC (30–45 Hz) occurred in both small and large dynamic force conditions without any significant difference between both conditions. Although in some subjects beta-range CMC was observed during both dynamic force conditions no significant difference between conditions could be detected. With respect to the motor performance, the lowest errors were obtained in the static force condition and the highest ones in the dynamic condition with large amplitude. However, when we normalized the magnitude of the errors to the amplitude of the applied force (relative errors) no significant difference between both dynamic conditions was observed.

**Conclusion:**

These findings confirm that during dynamic force output the corticomuscular network oscillates at gamma frequencies. Moreover, we show that amplitude modulation of dynamic force has no effect on the gamma CMC in the low force range investigated. We suggest that gamma CMC is rather associated with the internal state of the sensorimotor system as supported by the unchanged relative error between both dynamic conditions.

## Background

Synchronization between neurons in motor cortex and motor units has been extensively investigated since the early 90s. A major break through was a study on monkeys showing that cortical and motor oscillations were synchronized with the motor units of the contralateral muscles [2]. Conway *et al.*[3] showed for first time this mechanism in humans: Using one MEG channel they recorded the cortical motor activity and the surface electromyogram (EMG) of a contralateral active muscle during maintained voluntary contraction. Applying coherence analysis, these authors demonstrated coherence between cortical rhythms and rectified EMG confined to the beta (15–30 Hz) frequency range. They interpreted this beta-range synchronization as evidence for the involvement of the cortical motor neurons in the motor units' synchronization.

For the last ten years, research on the beta-range CMC has shown that it is task dependent [4,5], that it reflects attention and precision [6,7], compliance of the gripped objects [8,9], displacement [10], magnitude of force [11] and learning processes [12]. A major step towards understanding the functional significance of the beta-range CMC during steady-state force was provided only recently: Kristeva, Patino & Omlor [13] demonstrated that the CMC correlates with motor performance, namely that increased beta-range CMC improves motor performance. Therefore, the authors suggested that the beta-range CMC reflects effective corticospinal interaction.

Although the studies mentioned above have extended our knowledge about motor control, the investigation of the CMC was restricted mainly to steady-state motor output (isometric contraction and precision grip). This contrasts with the existence of many dynamic processes in daily life, which generally require constant adaptation of the motor outputs. Moreover, it has been suggested that static and dynamic force can be controlled by fundamentally different processes within the central nervous system [14]. These arguments call for the investigation of CMC under dynamic condition. In a recent study of us the EEG-EMG coherence during a steady-state motor output was compared to a periodically modulated dynamic isometric force output [1]. In the static condition, significant coherence was confined to the beta-range. In the dynamic condition, the most distinct coherence occurred in the gamma-range and the significant beta-range coherence was strikingly reduced. We concluded that during dynamic force the corticospinal oscillation mode shifts towards higher (principally gamma) frequencies for the rapid integration of the visual, somatosensory and cognitive information required to produce the appropriate motor command.

The aim of the present study was to investigate the modulation of the CMC by the amplitude of the periodically modulated dynamic force. Two dynamic force amplitudes were selected for this purpose. The periodically modulated target force, which had to be compensated isometrically by the subject, had the same frequency but different amplitudes. Static force was used as the control condition. Additionally, we investigated cortical spectral power (SP) and the motor performance during all three conditions.

We confirmed our previous result [1] that CMC is predominantly confined to gamma-range during dynamic force condition. Moreover, we show that the gamma-range CMC during dynamic force output is amplitude independent, *i.e*. that gamma-range CMC is not modulated by changes in target force amplitude, at least for the range of amplitude modulation and level of force investigated.

## Results

### Behavioral performance

All subjects performed the task according to the instructions. None of the subjects reported fatigue during the experimental session.

**Figure 1:**
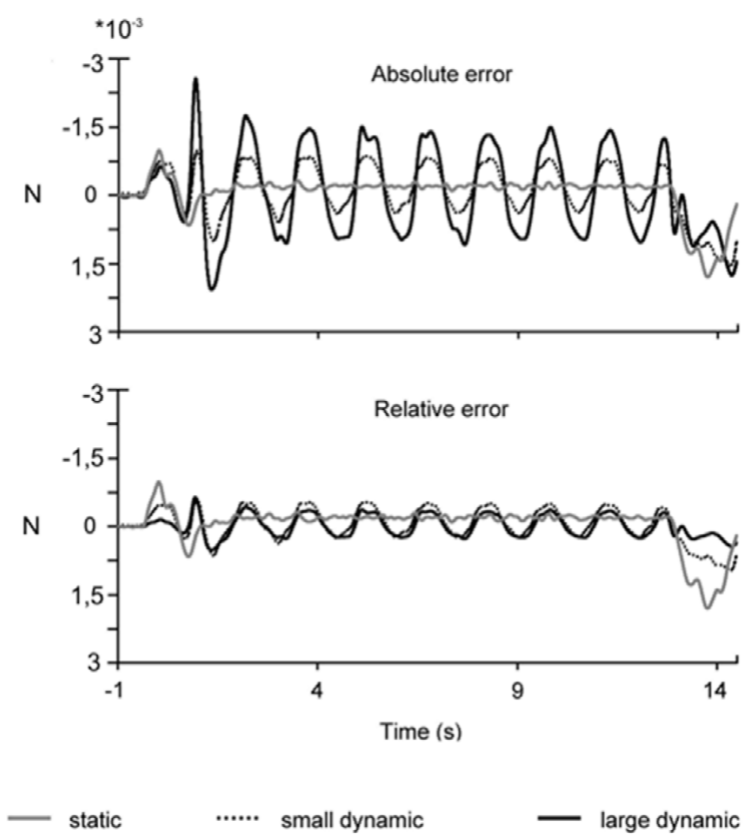
**Performance Analysis**. Motor performance error, upper panel absolute error, lower panel relative error (the error after normalization to the amplitude of the force) for 8 subjects (Grand average). The static force (*SF*) condition is marked as grey line, the small dynamic (*sD*) condition as dotted black line and large dynamic (*lD*) condition as thick black line.

The lowest absolute performance error was observed in the static force condition (*SF*) and the highest one in the large dynamic (*lD*) force condition. The absolute performance differences between the three conditions were statistically significant (p = 0.002, Friedman test, n = 8 throughout the whole manuscript). In detail, the pair-wise comparisons yielded statistical significance between *SF *than small dynamic (*sD*) condition (p = 0.015, Wilcoxon test; n = 8 throughout the whole manuscript), between *SF *and *lD *condition (p = 0.007, Wilcoxon test), and between *sD *and *lD *condition (p = 0.0234, Wilcoxon test). The mean absolute performance error across all 8 subjects is shown in Fig. 1 (upper panel).

However, no significant difference between both dynamic conditions was observed for the relative performance errors (p = 0.742, Wilcoxon test; Fig. 1, lower panel).

### Corticomuscular coherence (CMC)

**Figure 2:**
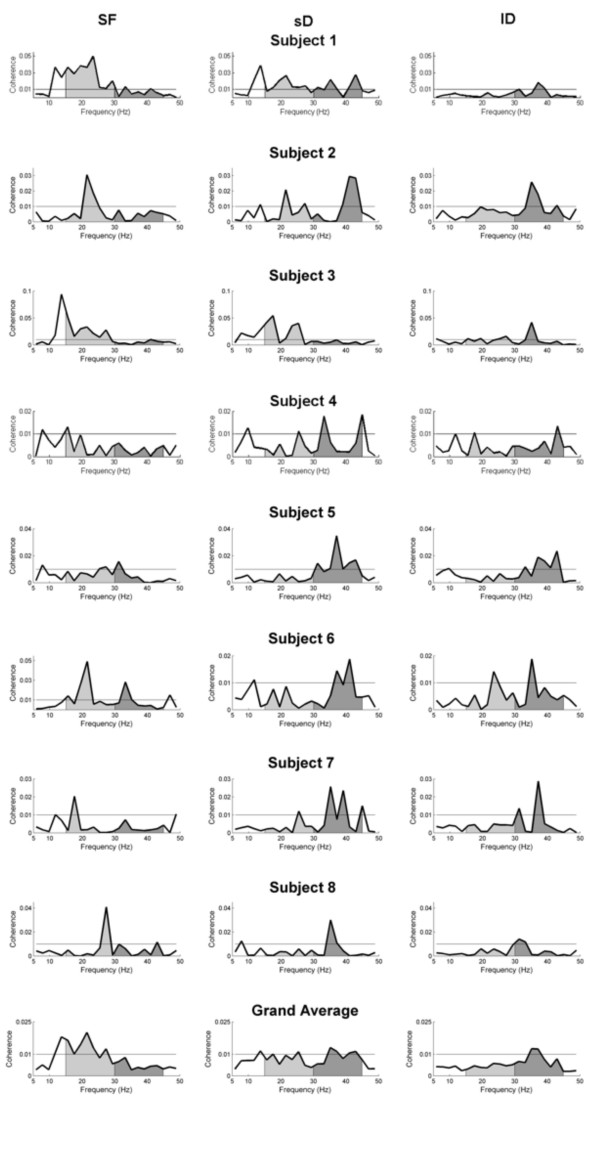
**Frequency-coherence plots**. Frequency-coherence plots for EEG-EMG coherence during static force (*SF*) condition (left panel) during small dynamic (*sD*) condition (middle panel) and large dynamic (*lD*) condition (right panel) for single subjects and as grand average. The beta-range (15–30 Hz) is marked in light grey, the gamma-range (30–45 Hz) in dark grey. Note that during the *SF *condition the most prominent activity occurs in the beta-range. During both dynamic conditions previous beta-range coherence is decreased and the general activity is shifted towards high frequencies, principally in gamma-range. During *sD *condition remains significant beta-range CMC, which is absent during *lD *condition.

Significant broad-band coherence during *SF *and during both dynamic conditions was observed. For subjects 1 to 6, coherence was most pronounced over the left motor cortex contralateral to the active right index finger (EEG channels C3, C1, FC3 and FC1) with the flexor digitorum superficialis and, for subjects 7 and 8, with the first dorsal interosseus. CMC during *SF *output was observed as expected in the beta-band. During both dynamic conditions, CMC was principally in the gamma band, which confirms our previous findings [1]. Inter-individual differences were however found. Figure 2 displays original curves of CMC in the three conditions (*SF*, *sD *and *lD*), as well as the grand average for all eight subjects.

Subject 2, representative of the majority of the subjects (6 out of the 8), showed as expected beta-range CMC with a maximum peak at 21.5 Hz in the *SF *condition. In the *sD *force condition, CMC was observed predominantly in the gamma-range (41 Hz), with some significant beta-range CMC which was however lower than in the *SF *condition. In the *lD *condition, only a prominent gamma-range CMC peak at 35.2 Hz was observed.

Two other subjects (S3 and S5) displayed quite different patterns. Subject 3 had CMC at higher beta frequencies during *sD *as compared to *SF *and, in the *lD *condition, CMC predominantly in the gamma-range without any beta. It is important to note that for this subject during the *SF *output the predominant peak occurred at 13.7 Hz which was reduced in *sD *condition. The second subject with a different CMC pattern (S5) showed during *SF *condition CMC in high beta and low gamma band. During both, *sD *and *lD *conditions, the main CMC occurred at higher frequencies in the gamma band.

When the results were pooled for all subjects (lowest panel in Fig. 2) the following picture occurred: In the *SF *condition a significant broad-band beta CMC was observed with maximum amplitude ranging from 0.012 to 0.051 across subjects. In the *sD *condition, the most prominent CMC occurred in the gamma-range with two predominant peaks at 35.2 and 43 Hz. This gamma-band CMC yielded amplitude values from 0.018 to 0.035. One significant peak also remained in beta-range at 25.4 Hz but was smaller than in the *SF *condition (p = 0.0156, Wilcoxon test). In the *lD *condition, only one significant CMC peak in the gamma-band (35.2 Hz) was obtained with an amplitude in the range from 0.013 to 0.042. The beta-range CMC decreased significantly compared to that in the *SF *condition (p = 0.0078, Wilcoxon test).

**Figure 3:**
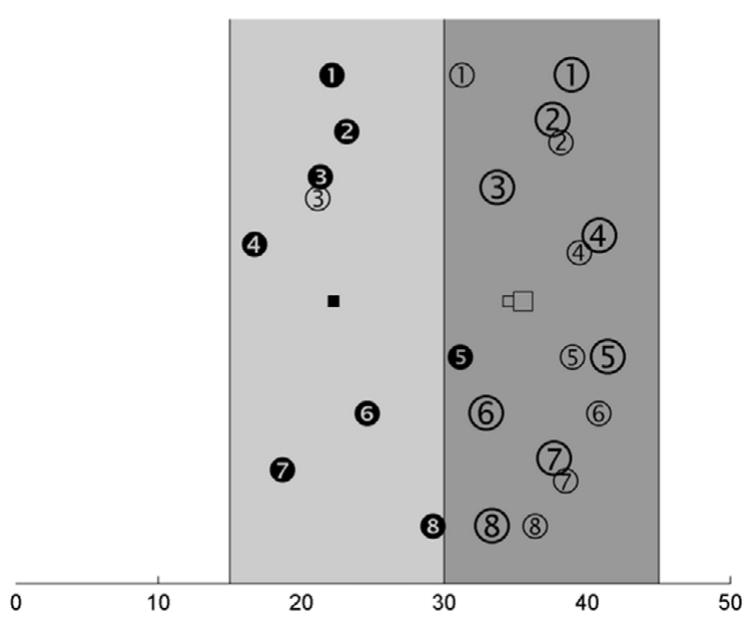
**Center of gravity for frequency**. (a) Individual frequency values of maximum coherence (centre of gravity) calculated for the whole range study (15–45 Hz). Each subject is represented by a number. Filled circles and square: individual values and their mean for the SF condition. Small empty circles and square: values and mean for the *sD *condition. Large empty circles and square: values and mean for the *lD *condition. The beta-range (15–45 Hz) is marked in light grey, the gamma-range (30–45 Hz) in dark grey. Note that the frequency values for the both dynamic conditions are systematically higher than for the static condition, but there are no differences of the frequency values between both dynamic conditions.

In general terms, we observed a shift of the CMC from lower to higher frequencies (principally from beta to gamma) when comparing static and dynamic conditions (p = 0.0098, Friedman test) which is in accordance with our previous study [1]. This shift is best visualized on the display of the individual CMC centres of gravity (Fig. 3).

In 6 subjects, the centre of gravity was shifted from beta- to gamma-range, between the *SF *and *sD *condition, the extent of this shift varying between 7 and 23 Hz. In subject S3 we did not obtain any shift over the frequency range 15–45 Hz and subject 5 showed the shift within gamma-range. The CMC frequency shift between *SF *and *lD *force occurred in all subjects and ranged from 7 to 24 Hz. The difference in the dominant frequencies of the CMC among the three conditions was statistically significant (p = 0.0098, Friedman test). The shifts to higher frequencies between *SF *and *sD *as well as between *SF *and *lD *were highly significant (p = 0.0156 and p = 0.0078 respectively, Wilcoxon test). In contrast, we did not find any statistically significant difference in CMC frequency between both dynamic force conditions (p = 0.9453, Wilcoxon test).

### Cortical motor spectral power (SP)

**Figure 4:**
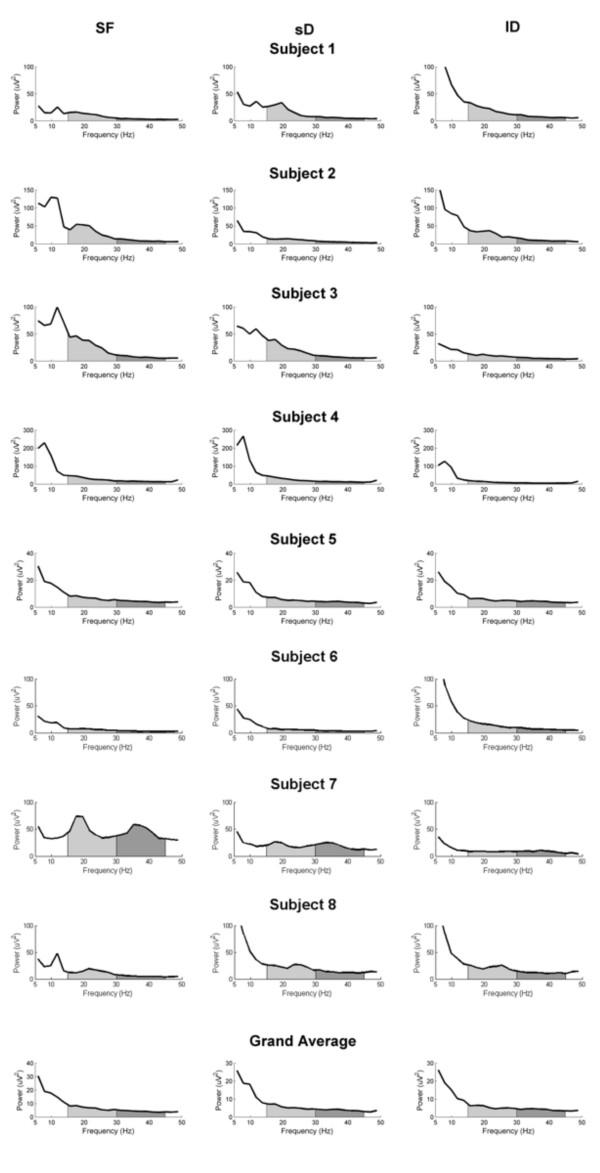
**EEG spectral power plots**. EEG spectral power during *SF *output (left panel), during *sD *force output (middle panel) and during *lD *force output (right panel) for single subjects and as grand average. The beta-range (15–30 Hz) is marked in light grey. The gamma-range (30–45 Hz) is marked in dark grey.

We calculated EEG spectral power for the channel of maximal coherence. Fig. 4 shows the EEG spectral power for all subjects investigated and the grand average. We observed a beta-range SP decrease in six out of the eight subjects investigated (S2, 3, 4, 5, 6, 7) in *sD *condition and in five subjects (S2, 3, 4, 5, 7) in *lD *condition, as compared to *SF*. However, the beta- and gamma-range SP differences among the three conditions were not statistically significant.

### EMG spectral power

**Figure 5:**
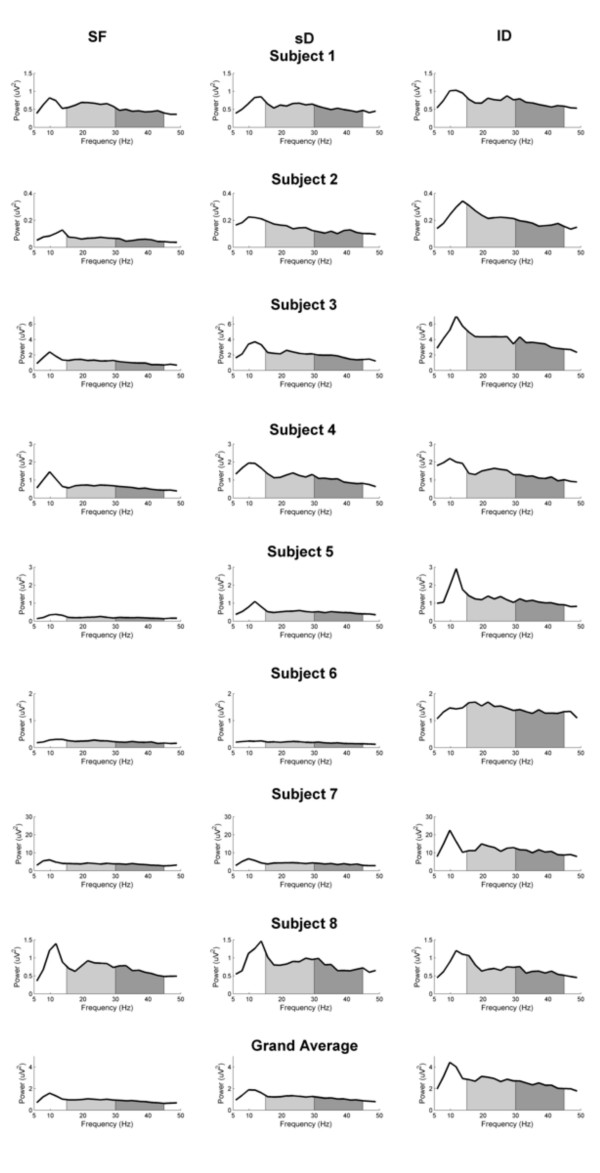
**EMG spectral power plots**. EMG spectral power during *SF *output (left panel), during *sD *force output (middle panel) and during *lD *force output (right panel) for single subjects and as grand average. The beta-range (15–30 Hz) is marked in light grey. The gamma-range (30–45 Hz) is marked in dark grey. Note the highest EMG SP during *lD *condition.

Fig. 5 shows the EMG spectral power data. We observed statistically significant differences among all three conditions in both frequency ranges, beta (p = 0.0208 Friedman test) and gamma (p = 0.0111, Friedman test). The highest EMG SP was observed during *lD *condition and the lowest EMG SP during *SF *(Fig. 5). The paired Wilcoxon test revealed significant differences for both frequency ranges in the comparisons between *SF *and *sD *(beta p = 0.0391, gamma p = 0.0156), between *SF *and *lD *(beta p = 0.0156, gamma p = 0.0156), and between *sD *and *lD *(beta p = 0.0391, gamma p = 0.0234).

## Discussion

The main goal of the present study was to determine whether the amplitude modulation of dynamic force output has an influence on the CMC. Our results show that the frequency range of synchronization and the amount of CMC during the two dynamic conditions with different amplitudes are similar. Consistent with our previous work [1] we found a CMC shift from lower frequency (beta) during static force output to higher frequency (principally gamma) during dynamic force output.

### Comparison of motor performance between SF, sD and lD

Slobounov, Hallett & Newell [15] hypothesize that the increase in performance error corresponds to task difficulty. In our case, the lowest absolute error was during *SF *condition and therefore, we conclude that *SF *was the easiest task to control and perform. The finding that the error in *lD *is significantly higher than in *sD *suggests that large dynamic force condition has a higher level of difficulty. However, the similar relative errors indicate that *sD *and *lD *conditions were controlled by the subjects in a similar way. This means that the two dynamic conditions have the same level of difficulty for the subjects.

### Cortical and spinal neurons are synchronized at gamma frequency during dynamic force conditions

Our results clearly show that during isometric compensation for a periodically modulated force with two amplitudes the CMC is in a similar frequency range, predominantly at gamma frequencies, and also occurs with similar amplitude. This demonstrates that the amplitude of dynamic force does not modulate the CMC. The following aspects make both dynamic conditions similar: (*i*) Both dynamic forces have a similar temporal pattern, as the modulation frequency is the same. (*ii*) Thus, both dynamic tasks require the same level of prediction and therefore, readiness to respond should also be the same. (*iii*) Thus, we can hypothesize that both *sD *and *lD *conditions required from the subjects a similar level of attention. (*iiii*) If the amount of attention and prediction are similar, the complexity of processing involved in motor planning should be also similar [16].

During both dynamic conditions, neurons in motor cortex and spinal motoneurones are synchronized at higher frequency (for 7 out of the 8 subjects it was gamma frequency range) compared to the static force condition. Moreover, the degree of this shift was the same for both dynamic conditions. Therefore, we conclude that both *sD *and *lD *force conditions require the same level of sensorimotor and visual integration. This is well in line with the similar motor performance in both dynamic conditions, revealed by the similar relative errors.

Taking the frequency range of CMC into account, we found large inter-individual differences for both beta- and gamma-band (Fig. 3). This is the reason for the low strength of CMC in the grand average (Fig. 2). These large inter-individual differences in the beta-range correspond to previous results in humans [17] and monkeys [4]. The large inter-individual differences in the gamma-range also are in accordance to our previous findings [1]. These present data support the assumption that frequency and strength of CMC are characteristic for every single subject.

Our findings about EEG spectral power confirm the complicated relationship between spectral power and CMC described by others [18] and ourself [1,13]. In this respect, it is important to note that the CMC increase in our experiment is not associated with higher EEG power. For example subject 7 in Fig. 4 shows in *SF *high SP in beta and gamma range while the CMC in this condition was restricted to beta range. The clarification of this relationship surely requires further exploration, *e.g*. using electrocorticogram.

### Possible function of gamma CMC activity

A number of previous studies suggest that the gamma-range CMC could be the consequence of greater attention to the task [1,11,19,20] and that it is connected to the readiness to respond [21]. In [1] we showed that during the control of a more complex task like our visuomotor dynamic force modulation the sensorimotor system is synchronized at gamma frequencies to rapidly integrate visual, proprioceptive, tactile and cognitive (prediction and planning) information.

An investigation on the deafferented patient GL showed that the proprioceptive feedback is mandatory for the genesis of gamma-range CMC [22]. Our present observation that the amplitude of the dynamic force does not modulate the CMC suggests that changes in proprioceptive input during dynamic forces in the range from 1.6 to 4% MVC were not distinct enough for this modulation.

## Conclusion

To conclude, during the control of dynamic forces, the brain engages into a neural state characterized by synchronization at gamma range to rapidly integrate visual, prioprioceptive, tactile and cognitive information and timely recalibrate the motor system to generate the required force, independently of its magnitude.

## Methods

### Subjects

Eight healthy volunteers (mean age 28 ± 13 years, 2 men) without any history of neurological disease participated in the study. All subjects were right handed according to the modified Edinburgh Handedness Inventory [23]. They gave written consent to the experiment in accordance with the declaration of Helsinki and all procedures were approved by the local ethics committee.

### Paradigm

**Figure 6:**
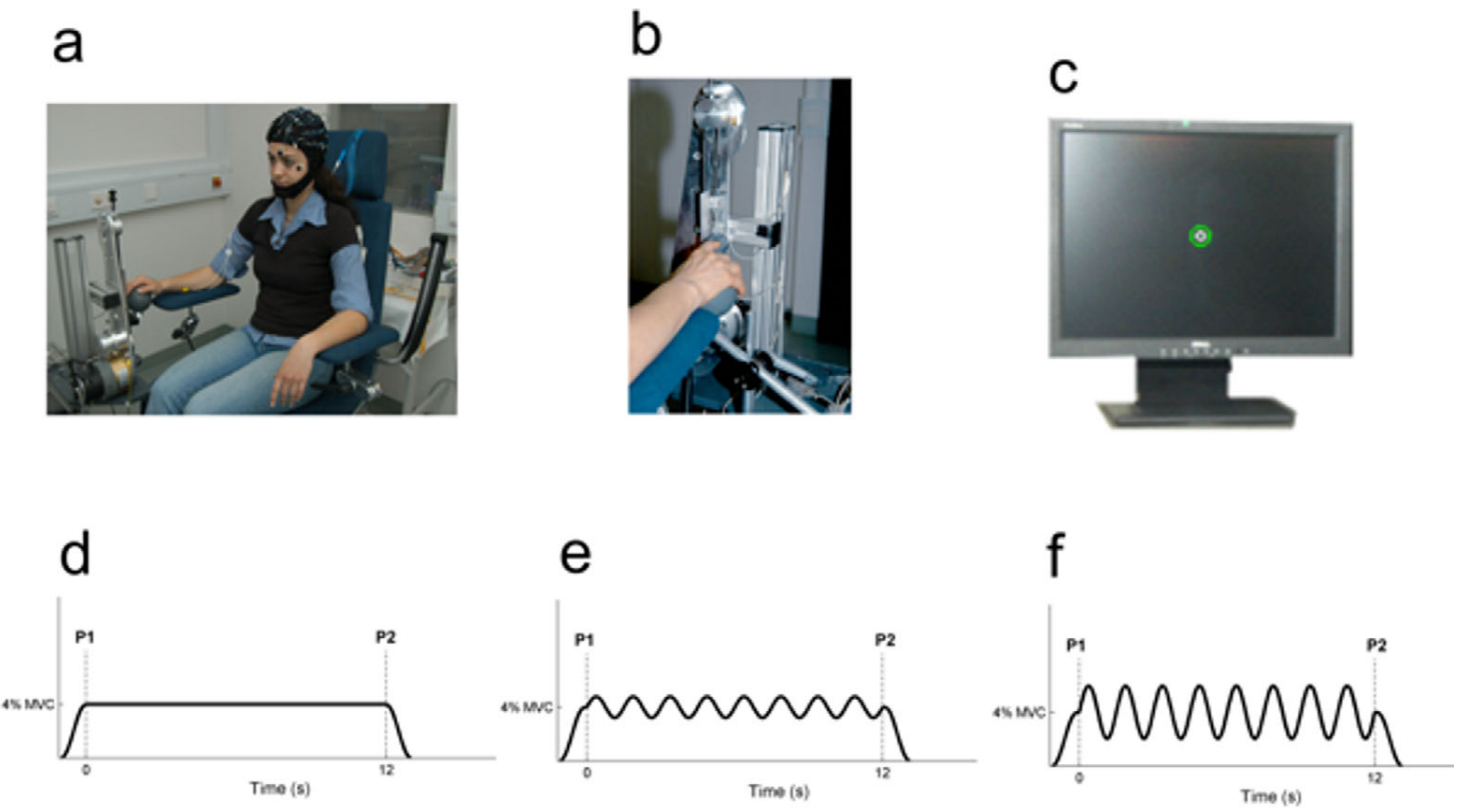
**Experimental setup**. (a) High-resolution EEG recorded from 48 scalp positions together with the electrooculogram (EOG) and the EMG. (b) Manipulandum. (c) Visual feedback about the position of the ring displayed on a monitor in front of the subject. (d) Force profile generated by the manipulandum during static (*SF*), (e) small dynamic (*sD*) and (f) large dynamic (*lD*) condition. After a gradual increase of force to 4% MVC, the subject has to maintain the ring in its central position for 12 seconds. Both dynamic conditions have a superimposed sinusoidal function.

During the experimental session, the subject sat in an electrically shielded, dimly lit room. The right arm was supported by a splint and the subject was instructed to place the hand over a sphere, and the right index finger in the ring of a home-made manipulandum (*cf*. Fig. 6a, b).

The manipulandum was designed for applying vertical forces on the finger, at the level of the metacarpophalangial joint. A computer-controlled tooth belt drive produced a variable upwards force on the ring. The force generated by the manipulandum was called target force (TF). The subject had to compensate the force generated by the manipulandum isometrically and maintain the ring in its initial position. The force exerted by the subject was called exerted force (EF). Visual feedback about the position of the ring was provided to the subject via a monitor 60 cm in front of him/her with two circles (Fig. 6c). The moving white inner circle (radius 2.5 mm) represented the position of the ring along the vertical axis, while the green outer circle (radius 7.5 mm) was fixed and represented the ring's reference position. The visual feedback of the actual displacement of the ring was enhanced, so that 1 mm of distance traveled by the ring was represented by 2.8 mm traveled by the white circle on the monitor. Then, the instruction given to the subject was to exert a compensating force, in order to keep the small white circle inside the green circle.

Three different experimental conditions were investigated in a given recording session:

• *Static force (SF) condition: *The manipulandum generated a steady force at 4% of the maximum voluntary contraction (MVC) determined prior to the experiment (cf. Fig. 6d).

• *Small dynamic (sD) force condition: *During this condition, the manipulandum generated a sinusoidal modulation of the 4% MVC force at frequency of 0.7 Hz and with peak-to-peak amplitude of 1.6% MVC (cf. Fig. 6e).

• *Large dynamic (lD) force condition: *During this condition, the manipulandum generated a sinusoidal modulation of the 4% MVC force at frequency of 0.7 Hz and with peak-to-peak amplitude of 4% MVC (cf. Fig. 6f).

Two of the conditions (*SF *and *sD*) were the same as in [1]. The mean force level (4% MVC) was the same for all three conditions. Although an amplitude modulation of 2.5 times may not constitute a large step between "small" and "large" dynamic force condition we sought to investigate fine forces. This is based on findings that for finger isometric force the motor cortex activity is most sensitive to fine-graded low force under 10% MVC [24-26]. We therefore decided to use a force offset of 4% MVC which limits the range of peak-to-peak modulation.

To ensure a smooth start and end of the generated force by the manipulandum a rising cosine function with duration of 100 ms was used. After the increase of the force to 4% MVC, the subject had to keep the force required on the visual display, keeping the ring in its central position for 12 s. Subjects performed 35 trials for each condition. Rest intervals of approx. 10 s were inserted between trials. The subjects were instructed to avoid any other movements and to fix their gaze on the dysplay circle representing the position of the ring (the visual feedback during the task).

### Recordings

EEG (band pass 0–200 Hz, sampling rate 1000 Hz) was recorded from 48 scalp positions according to the extended 10–20 system (Synamp 2, NeuroScan, El Paso, TX, USA) referenced to Cz (Fig. 6a) with ground at FzA. Electrode impedances were under 5 kOhm. The EOG (same band pass and sampling rate as for EEG) was recorded to exclude trials contaminated with eye movements from further analysis. Electromyographic activity (EMG, band pass DC-200 Hz; sampling rate 1000 Hz) was recorded from the pars indicis of the right flexor digitorum superficialis muscle and from first dorsal interosseus (FDI). Both target force (TF) and exerted force (EF) were recorded in parallel with the electrophysiological data (same band pass and sampling rate as for EEG). EEG, EOG and EMG were stored and analyzed off-line.

Additionally each subject was given a short practice session prior to the experiment (5 trials for each condition) to become familiar with the behavioral task.

### Data analysis

#### EEG and EMG data processing

Data analysis was performed with the commercial software Brain Vision Analyzer (Brain Vision Version 1.05, Brain Products GmbH, Munich, Germany).

Continuous data between semi-automatically put markers (P1 and P2) (*cf*. Fig. 6d, e, f) was divided into successive non-overlapping segments of 512 ms length, allowing for a frequency resolution of 1.96 Hz. To avoid transient effects, data related to the initial and last force ramps phase were excluded from further analysis.

Artifact rejection was visually performed off-line trial-by-trial to exclude segments contaminated with eye movements. The EEG was then transformed into the reference free current source density distribution (CSD) which reflects the underlying cortical activity and removes nearly all volume conduction effects [27]. The CSD was computed using the spherical spline interpolation method [28] as implemented in the commercial software "Brain Vision 1.05."

For EMG data we applied a high pass filter of 5 Hz. Then EMG signal was rectified as it is known that full wave rectification provides the temporal pattern of grouped firing motor units [29]. The discrete 512 points Fourier transform was calculated for each segment for the whole 1 to 500 Hz frequency range. A total of 300 artifact-free segments were analyzed for each subject.

#### Calculation of the EEG-EMG coherence (CMC)

*Coherence values *(*Coh*) were calculated between the rectified EMG and the EEG channels in order to calculate the synchronization between the two signals. Coherence values (Coh) were calculated by the following formulae:

$$Coh_{c1,c2}\left(f\right)=\frac{{\left|S_{c1,c2}\left(f\right)\right|}^{2}}{\left|SP_{c1}\left(f\right)\right|\times \left|SP_{c2}\left(f\right)\right|}$$

where:

$$S_{c1,c2}\left(f\right)=\frac{1}{n}\sum_{i=1}^{n}C1_{i}\left(f\right)C2_{i}^{*}\left(f\right)$$

*S*_*c*1, *c*2_(*f*) is the cross-spectrum for the EEG (channel *c1*) and the rectified EMG (channel *c2*) at a given frequency *f*, and *SP*_*c*1_(*f*) and *SP*_*c*2_(*f*) are the respective power spectra for channels *c1 *and *c2 *at the same frequency *f*. *C1 *and *C2 *are the Fourier transformed data of channels *c1 *and *c2 *in a given segment number *i *(*i *= 1 ... *n*) and '*' indicates the complex conjugate.

Thus, the coherence value *Coh*_*c*1,*c*2_(*f*) corresponds to the squared magnitude of a complex correlation coefficient, and is a real number between 0 and 1.

Coherence values were considered not to occur by chance if the resulting values lie above the confidence level (*CL*) [30], where:

$$CL(\alpha )=1-{\left(1-\alpha \right)}^{\frac{1}{n-1}}$$

*n *is the number of segments and *α *is the desired level of confidence. We considered coherence to be significant above the 95% confidence limit.

First, coherence values were calculated between the rectified EMG and all EEG channels. Significant CMC values were observed over the left sensorimotor area contralateral to the right index finger movement (FC1; FC3; C1; C3; CP1 and CP3). Then, that EEG-EMG pair, where maximal coherence was observed in each subject was chosen for further analysis [1]. Maximum CMC was mostly localized over channels C3 and C1.

#### Calculation of the EEG and EMG spectral power (SP)

Power spectrum (SP) for a given channel (*c*) was further calculated according to the following equation

$$SP_{c}\left(f\right)=\frac{1}{n}\sum_{i=1}^{n}C_{i}\left(f\right)C_{i}^{*}\left(f\right)$$

where *C*_*i *_represents the Fourier transform of data segments *i *(*i *= 1 .... *n*) corresponding to channel *c*. '*' indicates the complex conjugate. EMG was rectified before SP analysis.

### Analysis of behavioral data

#### Computation of absolute error

Behavioral signals related to the task, the target force (*TF*), together with the force exerted by the subject (*EF*), were filtered off-line (band pass 0.5–30 Hz) to eliminate offsets accumulated during the recording. The 30 Hz high cut-off was selected to avoid power supply artifacts and was large enough to record the behavioral changes.

To evaluate the visuomotor performance, we computed error in force *E*, defined as the difference between the *TF *and the *EF:*

*E *= *TE *- *EF*

Since the force generated by the manipulandum (TF) was the reference to which the subject had to adapt, we calculated the mean squared error, *MSE*_*i*_, of the *EF *around its reference (*TF*) with the following formula:

$$MSE_{i}=\sum_{k=1}^{s}\frac{{\left(E_{k,i}\right)}^{2}}{s}$$

where again *i *= 1 ... *n *is the segment number, *k *= 1 ... *s *is the sampled point in the actual segment *i *and *s *= 512 is the number of sampled points in each segment.

Then we calculated the absolute cumulative mean squared error, *aMSE*, by adding up individual segment values

$$aMSE=\sum_{i=1}^{n}MSE_{i}$$

#### Computation of relative error

We normalized the magnitude of absolute cumulative errors *aMSE *to the amplitude of the target force.

$$rMSE=\frac{aMSE}{\left|TF\right|}$$

Whereas the |*TF*| for the *sD *was 1.6% MVC and for the lD was 4%MVC. Thus we obtained the relative error *rMSE *for both dynamic conditions.

### Statistical analysis

#### EEG-EMG coherence (CMC), EEG and EMG spectral power (SP)

To test for any statistical difference on CMC, cortical and muscles power between the *SF *and both dynamic conditions, we measured the area under the coherence curve and above the significance level A_coh_, and under the spectral power curve A_pow_, in two frequency-windows: 15–30 Hz for the beta-range and 30–45 Hz for the gamma-range.

Individual values for the area of the coherence were firstly transformed logarithmically to yield symmetric distributions according to the formula

*A'*_*coh *_= *log*(0.001 - *A*_*coh*_)

To evaluate the relation in magnitude between beta- and gamma-range coherence, we subtracted significant area corresponding to the gamma-range from the coherent area in beta-range.

*A *= *A'*_*coh*(*beta*) _- *A'*_*coh*(*gamma*)_

Afterwards the non-parametric Friedman test was applied to compare values A for CMC and A_pow _for SP measured in all 3 conditions for each single subject with the null hypotheses that the distributions of the values tested are the same across all 3 conditions. The Friedman test with the global null hypothesis was calculated first to avoid an alpha-adjustment in the simultaneous paired hypotheses. When the Friedman test indicated that not all of the three conditions were statistically equivalent, we performed a second non-parametric test (paired Wilcoxon test) on the resulting values A for CMC and A_pow _for spectral power. The null hypothesis was that the difference between the matched samples of coherence and power spectra arises from a distribution which is symmetric around zero. We applied this test on the following pairs: *SF *and *sD *condition, *SF *and *lD *condition and *sD *and *lD *condition. We used a third window between 15–45 Hz to evaluate the whole beta- and gamma-range activity together and calculated its centre of gravity (CoG, *i.e*. the frequency at which all CMC activity from 15–45 Hz could in theory be concentrated; around this frequency point, the CMC is balanced, *i.e*. is the same on the right and on the left). This was done according to:

$$CoG=\frac{\sum_{s=1}^{n}f_{s}*C_{s}}{\sum C_{s}}$$

where *s *= 1 ... *n *indicates the number of significant bins with its respective frequency value *f *and coherence amplitude *C*. We applied the Friedman test on the frequency values obtained from the centre of gravity for *SF*, *sD *and *lD *conditions to check whether there are any significant frequency shifts between all three conditions. We used paired Wilcoxon test on these frequency values for the same condition pairs as mentioned above.

#### Behavioral performance

To account for the inter-subject variability and to symmetrize the distribution, values corresponding to the behavioral performance were first logarithmically transformed:

*aMSE' *= *log*(*aMSE*)

The Friedman test was applied to check differences between all three conditions in terms of absolute performance errors. Afterwards we tested single pairs (*SF-sD, SF-lD, sD-lD*) using paired Wilcoxon test. The statistical difference in relative performance error between both dynamic conditions *sD*-*lD *was tested as well as using paired Wilcoxon test. A probability P value of less than 0.05 was considered significant for both statistical tests.

## Abbreviations

*aMSE *absolute cumulative mean squared performance error

*CMC *corticomuscular coherence

*E *error in force

*EEG *electroencephalography

*EF *exerted force

*EOG *electrooculogram

*EMG *electromyography

*FFT *Fast Fourier Transformation

*lD *large dynamic force output condition

*MVC *maximum voluntary contraction

*rMSE *relative cumulative mean squared performance error

*sD *small dynamic force output condition

*SF *static force output condition

*SP *spectral power

*TF *target force

## Authors' contributions

AA carried out data acquisition, data analysis and interpretation and participated in writing the manuscript. LP participated in the design of the study, in data analysis and interpretation and writing the manuscript. J-RN participated in the design of the experiment, data analysis and interpretation and revised the manuscript. MW assisted in data acquisition, data analysis and participated in writing and revision of the manuscript. MCHR participated in the study design, interpretation of the data and revised the manuscript. RK conceived the study and participated in all the steps of the realization of the study.

All authors read and approved the final version of the manuscript.
